# miR-145-3p Inhibits MuSCs Proliferation and Mitochondria Mass via Targeting MYBL1 in Jianzhou Big-Eared Goats

**DOI:** 10.3390/ijms24098341

**Published:** 2023-05-06

**Authors:** Emmanuel Odame, Li Li, Joshua Abdulai Nabilla, He Cai, Miao Xiao, Jiangfeng Ye, Yuan Chen, Bismark Kyei, Dinghui Dai, Siyuan Zhan, Jiaxue Cao, Jiazhong Guo, Tao Zhong, Linjie Wang, Hongping Zhang

**Affiliations:** Farm Animal Genetic Resources Exploration and Innovation Key Laboratory of Sichuan Province, College of Animal Science and Technology, Sichuan Agricultural University, Chengdu 611130, China

**Keywords:** miR-145-3p, MYBL1, CDR1as, COX1, COX3, myogenesis, mitochondria mass, goat

## Abstract

Muscle growth and injury-induced regeneration are controlled by skeletal muscle satellite cells (MuSCs) through myogenesis in postnatal animals. Meanwhile, myogenesis is accompanied by mitochondrial function and enzyme activity. Nevertheless, the underlying molecular mechanisms involving non-coding RNAs including circular RNAs (circRNAs) and microRNAs (miRNAs) remain largely unsolved. Here, we explored the myogenic roles of miR-145-3p and MYBL1 on muscle development and mitochondrial mass. We noticed that overexpression of miR-145-3p inhibited MuSCs proliferation and reduced the number of viable cells. Meanwhile, deficiency of miR-145-3p caused by LNAantimiR-145-3p or an inhibitor retarded the differentiation of MuSCs. miR-145-3p altered the mitochondrial mass in MuSCs. Moreover, miR-145-3p targeted and negatively regulated the expression of CDR1as and MYBL1. The knockdown of the MYBL1 using ASO-2′MOE modification simulated the inhibitory function of miR-145-3p on cell proliferation. Additionally, MYBL1 mediated the regulation of miR-145-3p on Vexin, VCPIP1, COX1, COX2, and Pax7. These imply that CDR1as/miR-145-3p/MYBL1/COX1, COX2, VCPIP1/Vexin expression at least partly results in a reduction in mitochondrial mass and MuSCs proliferation. These novel findings confirm the importance of mitochondrial mass during myogenesis and the boosting of muscle/meat development in mammals.

## 1. Introduction

Postnatal muscle growth and regeneration are governed by skeletal muscle satellite cell (MuSC)-derived myoblasts through myogenesis [1]. MuSCs are quiescently located between the plasma membranes of myofibers [2] and activated to generate multi-nucleated myotubes once muscle damage occurs [3,4]. That is, the myoblasts differentiate into mononucleated myocytes which then fuse to form multi-nucleated myotubes at the late stage of differentiation. Except for some well-known myogenic genes such as myogenic Factor 5 (Myf5), myogenic Differentiation 1 (MyoD), myogenin (MyoG) and myocyte enhancer factor 2C (MEF2), MYBL1 (also named A-myb) [5] is enriched in skeletal muscles [6]. Although previous reports suggest that MYBL1 inhibits differentiation, increases proliferation, and alters apoptosis in cells including hematopoietic cells, which appeared to be non-oncogenic [5,7,8,9], its function in myogenesis remains to be elucidated.

Additionally, non-coding RNAs such as microRNA (miRNA) and circular RNA (circRNA) play crucial regulatory roles during myogenesis, mainly through downregulating their targets [10,11]. For example, miR-145-3p, a passenger strand for miR-145-5p, enhances differentiation and inhibits proliferation in castration-resistant prostate cancer [12] and participates in goat muscle development [13], though the myogenic function of miR-145-3p has not yet been explored. circRNA CDR1as (cerebellar degeneration-related protein 1 antisense RNA, also termed as ciRS-7) is highly expressed during myogenesis and regulates muscle development [14,15,16] via serving as a competing RNA for muscle-related miRNAs, including miR-7, miR-206, and miR-671 [14,17]. Our previous research showed CDR1as/miR-7/insulin-like growth factor 1 (IGF1R) plays crucial roles in goat myogenesis [14].

Myogenesis is generally accompanied by mitochondrial biogenesis, function, and enzyme activity [18,19,20,21]; the mitochondria oxidative phosphorylation, mitochondrial DNA, and oxygen consumption rates increase along with myogenesis [18,22]. Differentiating myotubes rely on about 60% of mitochondrial respiration as their source of metabolic energy [23]. The cytochrome c oxidase (COX) genes, including COX1, COX2, and COX3 composing the core complex nuclear-encoded genes [24], are involved in the mitochondria respiratory chain complex IV assembly and the cellular respiratory mechanism, ion/electron transport, and other mechanisms that are involved in cellular growth and myogenesis [25,26]. Increased mitochondria activities upsurge the proliferation and differentiation of myoblasts [27] through upregulating myogenic regulatory factors, including Myf5, MyoD, MyoG, and myogenic regulatory factor 4 (MRF4), consequently promoting muscle growth and regeneration [11,28]. Alternatively, myogenic-induced transcriptional coactivators and factors regulate mitochondrial biogenesis [29,30,31]. Therefore, myogenesis progresses with mitochondrial biogenesis through this positive autoregulatory loop [32]. Hence, an intact and functioning mitochondrion is necessary for myogenesis [33,34].

The underlying molecular mechanisms connecting myogenesis and mitochondria function remain to be fully understood. Here, based on the RNA-seq data, we anchored miR-145-3p regulated by CDR1as in muscle development. Then, we explored the role of miR-145-3p and its target genes (MYBL1, COX1, and COX3) in myogenic proliferation, differentiation, and mitochondrial mass. These results provide further knowledge in expanding the roles of miR-145-3p, MYBL1, and CDR1as in goat myogenesis, which will contribute to how genes affect mitochondrial mass during myogenesis and the boosting of muscle/meat development in mammals.

## 2. Results

### 2.1. miR-145-3p Is Negatively Associated with Levels of MYBL1 mRNA in CDR1as-Perturbed Myocytes

**Figure 1:**
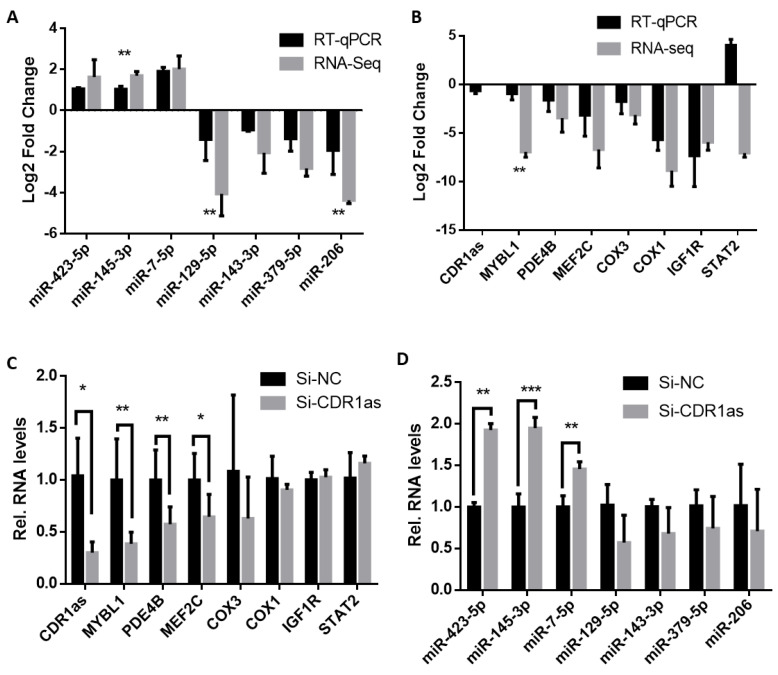
miR-145-3p is inversely related to MYBL1 in myogenic differentiation of CDR1as-disturbed MuSCs. A bar graph illustration of the comparison of relative fold changes (log2 fold change) of the RNA-seq and qRT-PCR of some selected DE upregulated and downregulated miRNAs, including (**A**) miR-145-3p, and (**B**) mRNAs, such as MYBL1 during CDR1as knockdown in myogenic differentiation. (**C**) Expression levels of MYBL1, other mRNAs, (**D**) miR-145-3p and other miRNAs during si-CDR1as. All experiments were repeated 3 times and statistical differences (*p* < 0.05) are differentiated by *, *p* < 0.01 by **, and *p* < 0.001 by ***. GAPDH (glyceraldehyde-3-phosphate dehydrogenase), was used as an internal control for the qRT-PCR. Data were analyzed by an unpaired 2-tailed *t*-test shown as mean ± SD with asterisks indicating the significance.

To explore gene networks affected by CDR1as in differentiating MuSCs, we performed RNA-seq as well as RT-qPCR and found that though some miRNAs like miR-206 are downregulated by CDR1as deficiency, a few miRNAs including miR-423-5p, miR-145-3p, and miR-7-5p are elevated during CDR1as knockdown (Figure 1A) [35]. Meanwhile, some well-known muscle genes including MEF2C, IGF1R, as well as PDE4B that are closely related to skeletal muscle proteolysis were down regulated by CDR1as deficiency (Figure 1B) [36]. Similarly, transcripts of cellular respiratory genes COX1, COX3. and proliferation-related MYBL1 [37] were lowered, suggesting that the miR-145-3p was inversely related to MYBL1 during CDR1as knockdown. Although, the altered expression trend from the RNA-seq was consistent with the qRT-PCR, there was a significant difference (*p* < 0.05) between the fold change from the RNA-seq and the qRT-PCR of some differentially expressed (DE) miRNAs (including miR-145-3p, miR129-5p, and miR-206) and DE mRNA (MYBL1) (Figure 1A,B).

We subsequently confirmed the endogenous relationship between miR-145-3p, MYBL1, and CDR1as during myogenesis. We quantified and found that CDR1as was highly expressed during MuSC differentiation, with the highest expression on day 5 (Supplementary Figure S2A). The myogenic proliferation genes, such as PCNA (proliferating cell nuclear antigen) and Pax7 (paired box 7), were highly expressed during proliferation, while differentiation markers MYHC (myosin heavy chain) and MyoG (myogenin) were highly expressed during differentiation (Supplementary Figure S2), confirming the stages of myogenesis (Supplementary Figure S2). Following that, CDR1as was knocked down in MuSCs, and the mRNA levels of MYBL1, PDE4B (Phosphodiesterase 4B), and MEF2C were downregulated, while those of some other mRNAs, including STAT2 and IGF1R, was insignificantly affected (*p* > 0.05) (Figure 1C). Additionally, the levels of miR-145-3p, miR-423-5p, and miR-7-5p were upregulated (*p* < 0.05) whilst those of miR-129-5p, miR-143-3p, miR-379-5p, and miR-206 were not significantly (*p* > 0.05) (Figure 1D). This confirms the ability of CDR1as to simultaneously regulate the expression of several miRNAs and mRNAs, including miR-145-3p and MYBL1, during myogenesis [35].

### 2.2. MYBL1 and CDR1as Are Targeted by miR-145-3p

**Figure 2:**
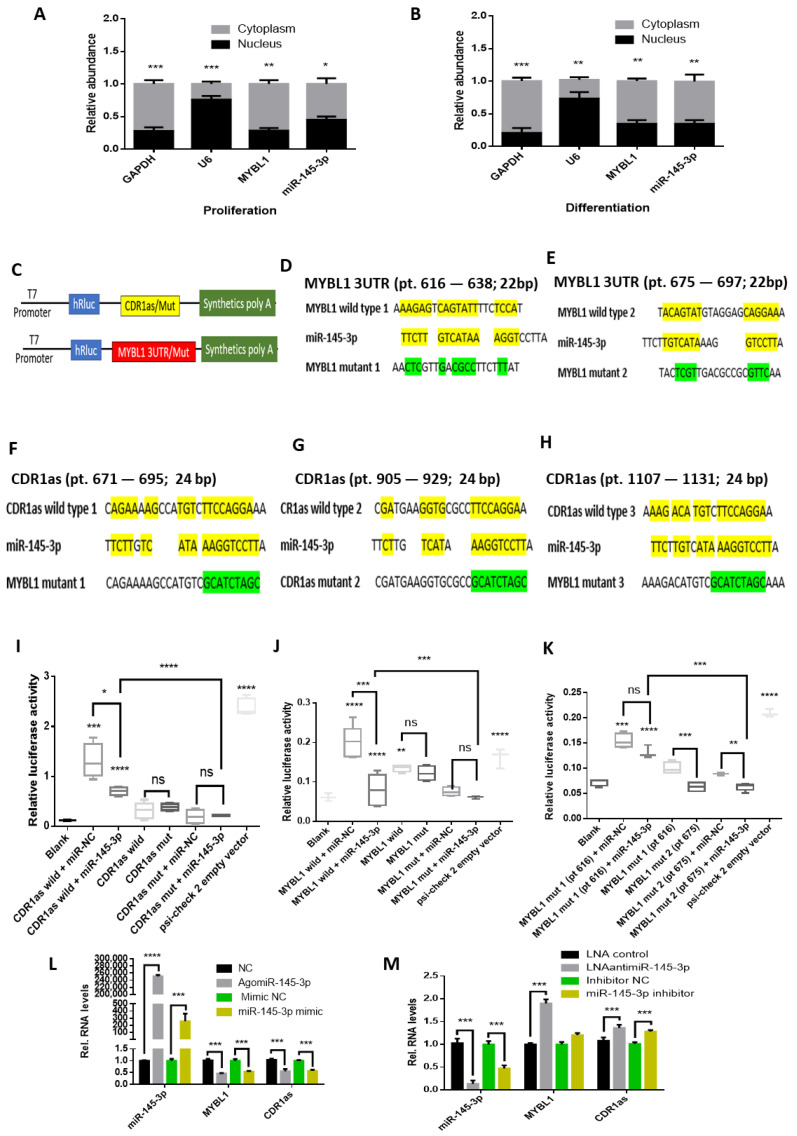
miR-145-3p binds to and negatively regulates MYBL1 and CDR1as transcripts in MuSCs. miR-145-3p is highly expressed in MuSCs cytoplasm; (**A**) the relative RNA levels of MYBL1 and miR-145-3p in proliferating (2 days) and (**B**) differentiating (4 days) MuSCs. GAPDH and U6 were used as control. All experiments were repeated three times and statistical differences (*p* < 0.05) are differentiated by *. (**C**) The binding sites of miR-145-3p on CDR1as and MYBL1 were cloned and/or mutated. The mutant or wild-type CDR1as and MYBL1 UTR constructs were placed between the hRluc and synthetic poly-A in the Psi-check 2 vector. (**D**–**H**) The binding sites of miR-145-3p on MYBL1 3 UTR at the nucleotide position 616, 675 and its mutated sites, and CDR1as and its mutated sites. The yellow highlights represent miR-145-3p binding sites on its target, while the green highlights represent those of the mutated sites on the target sequence. (**I**) The luciferase activity of MuSCs transfected with miR-145-3p mimic + CDR1as wild-type or mutant, (**J**) miR-145-3p mimic + MYBL1 wild-type or MYBL1 mutant, and (**K**) miR-145-3p mimic + MYBL1 mutant 1 or 2 were quantified using a microplate reader. Psi-check 2 was used as a negative control. The relative luciferase activity was calculated by Fluc/Rluc ratio. All experiments were repeated six times and statistical differences (*p* < 0.05) are differentiated by *, *p* < 0.01 by **, *p* < 0.001 by *** and *p* < 0.0001 by ****. The asterisks right above the box blots are the significant differences between the blank and the other vectors transfected used during the dual-luciferase experiment. The other comparisons made are indicated by the lines between each two different transfected vectors, which have the lines over them. The significance of means differences was analyzed by an unpaired 2-tailed *t*-test and presented as mean ± SD with asterisks indicating the significance. (**L**,**M**) The relative RNA expression levels of MYBL1 and CDR1as during miR-145-3p overexpression and knockdown in MuSCs.

The activities of miRNAs in the cytoplasm and nucleus have been reported [38,39,40]. We quantified the nuclear and cytoplasmic composition of miR-145-3p and MYBL1 in MuSCs. miR-145-3p and MYBL1 were highly expressed in the cytoplasm compared to the nucleus (Figure 2A,B). miR-145-3p potentially targeted several sites on CDR1as and MYBL1 3′ UTR, with a lower binding energy on CDR1as compared to those on MYBL1 (Supplementary Figure S3A,B) [41]. We employed a dual-luciferase reporter assay to confirm this miRNA-circRNA-mRNA binding prediction. Wild-type (wild) and mutant (Mut) reporter constructs (CDR1as and MYBL1 3UTR) (Figure 2C–H) were co-transfected with miR-145-3p mimics in proliferating MuSCs. There was a significant difference (*p* < 0.05) between the luciferase activity of cells transfected with miR-145-3p mimic + CDR1as wild-type or mutant, and miR-145-3p mimic + MYBL1 wild type, MYBL1 mutant, or mutant 1 or 2 (Figure 2I–K), confirming the binding of miR-145-3p on CDR1as and the MYBL1 3UTR. The luciferase activity of the CDR1as construct was higher than the MYBL1. However, the binding of miR-145-3p on MYBL1 3UTR was geared towards the nucleotide position 675 in MuSCs (Figure 2K).

We subsequently confirmed the regulatory effect of miR-145-3p on the expression levels of MYBL1 and CDR1as in MuSCs (Figure 2L,M). We used both miRNA mimics and AgomiRs to overexpress miR-145-3p while using LNAantimiR and miRNA inhibitors to inhibit its expression (details are in methods; 4.7 cell culture and treatments). Overexpressed miR-145-3p inhibited the expression levels of MYBL1 and CDR1as, while inhibiting miR-145-3p using either LNAantimiR and or miR-145-3p inhibitor reversed this inhibitory effect in proliferating MuSCs (Figure 2L,M). This confirms that miR-145-3p binds and inhibits the expression levels of MYBL1 and CDR1as in MuSCs during myogenesis.

### 2.3. miRNA-145-3p Inhibits Myogenic Proliferation, Differentiation, and Mitochondrial Mass

**Figure 3:**
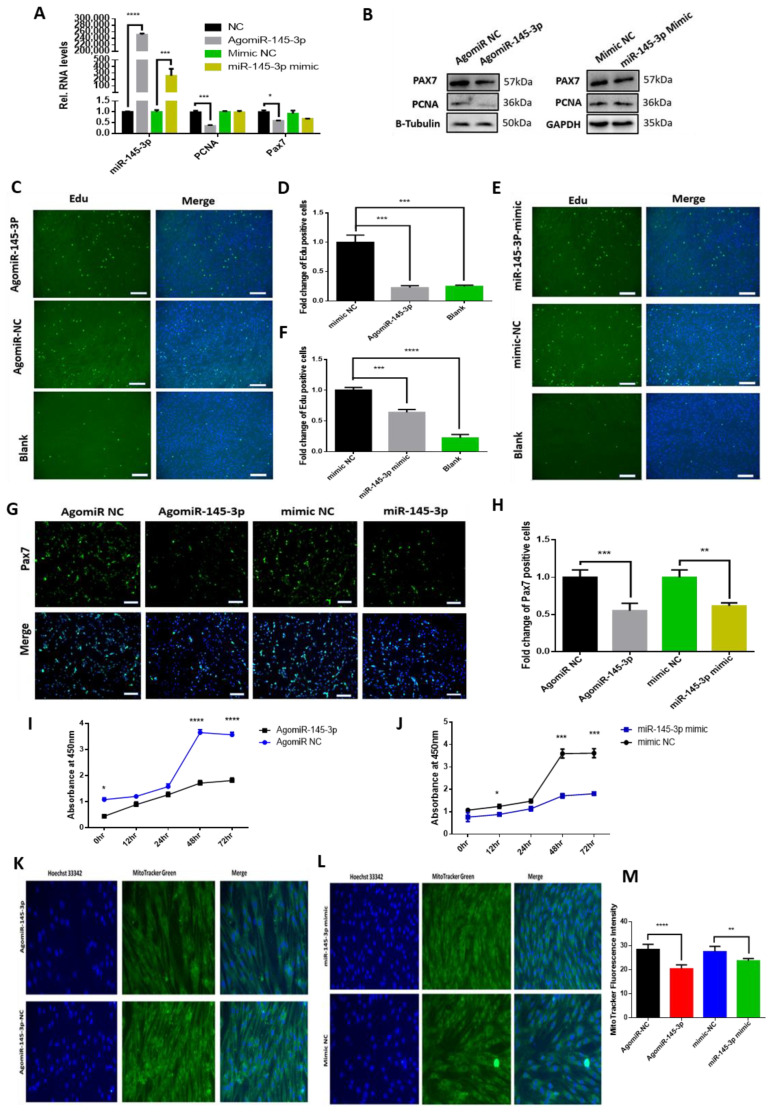
Ectopic miR-145-3p retarded myogenic proliferation and mitochondrial mass of goat MuSCs. MuSCs were transfected with either miR-145-3p mimic, AgomiR-145-3p, or mimic control, or substituted DMEM in place of the transfection medium (as a positive control). Overexpressed miR-145-3p inhibited the (**A**,**B**) expression of PCNA and Pax7. Subsequently, (**C**–**F**) cell nucleus was stained with Hoechst 33342 (blue), while proliferating cells were stained with EdU (green). Fold change of EdU-positive cells transfected with AgomiR-145-3p, mimic NC, and DMEM. (**G**) Overexpressing miR-145-3p using miR-145-3p mimics and AgomiR-145-3p inhibited Pax7 immunofluorescence in MuSCs. The cell nucleus was stained with DAPI (blue), while Pax7 protein was stained green. (**H**) Fold change of Pax7-positive cells transfected with miR-145-3p mimic, AgomiR-145-3p, and mimic NC. All experiments were repeated 3 times, and statistical differences (*p* < 0.05) are differentiated by *. (**I**) miR-145-3p reduces the number of viable cells during MuSC proliferation. The number of viable MuSCs during proliferation when transfected with miR-145-3p mimics or (**J**) AgomiR-145-3p. Statistical significance differences (*p* < 0.05) were represented with *, *p* < 0.01 by **, *p* < 0.001 by *** and *p* < 0.0001 by ****. (**K**–**M**) Overexpressing miR-145-3p inhibited the of mitochondrial mass in proliferating MuSCs. MuSCs were cultured, and the nucleus was stained with Hoechst 33342, while the mitochondria were stained with MitoTarcker green. Scale bar 50 µm.

We quantified the expression profile of miR-145-3p during myogenesis and found that miR-145-3p was highly expressed during differentiation (day 5) with lower expression levels during proliferation (Supplementary Figure S2). Moreover, overexpressing miR-145-3p in proliferating MuSCs inhibited the expression of proliferation markers PCNA and Pax7 (Figure 3A,B), and the number of EdU-positive cells (Figure 3C–F). Further, ectopic miR-145-3p strongly decreased the number of Pax7-positive cells (Figure 3G,H). Overexpressed miR-145-3p reduced the number of viable cells, while knockdown reduced this effect (Figure 3I,J, Supplementary Figure S4A,B). Since myogenesis is accompanied by mitochondrial biogenesis, which supplies the cells with energy for cellular processes [18,19,20,21], we valuated mitochondrial mass using MitoTarcker green and found that overexpressed miR-145-3p inhibited the mitochondrial mass in proliferating MuSCs (*p* < 0.05) (Figure 3K,L, Supplementary Figure S4C–E). Conclusively, miR-145-3p inhibits MuSCs proliferation, presented as decreased levels of proliferation markers Pax7 and PCNA; the number of EdU-positive cells; viable cells; and mitochondrial mass.

**Figure 4:**
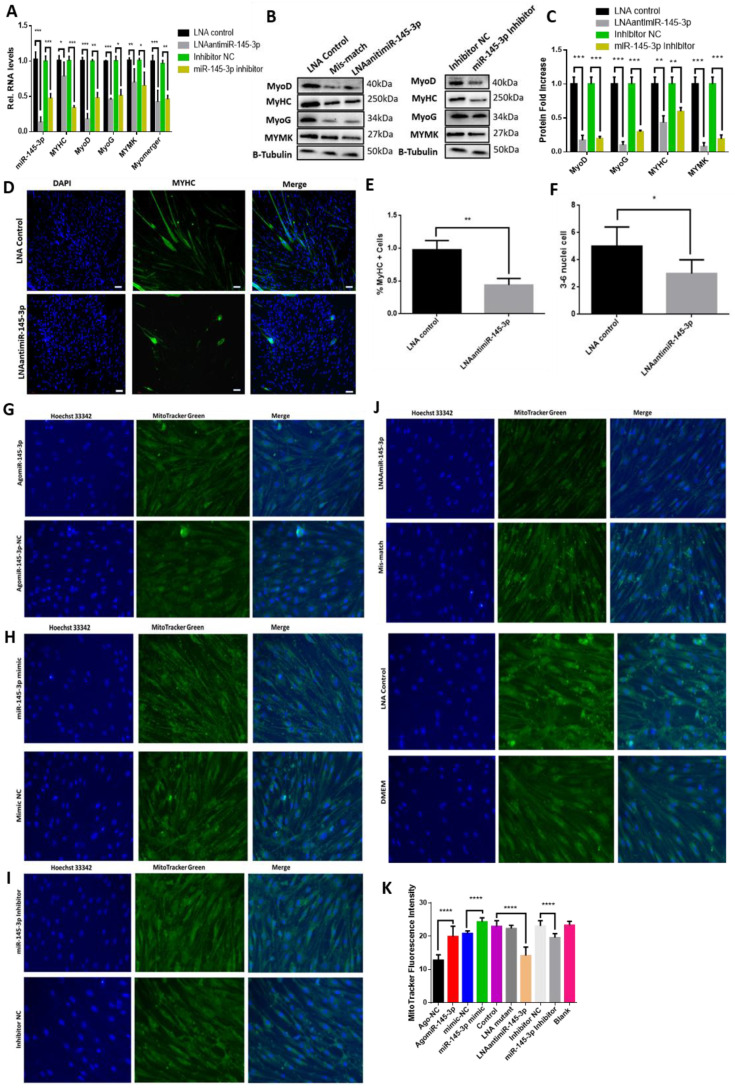
Deficiency of miR-145-3p inhibits myogenic differentiation and mitochondria mass. (**A**–**C**) Knockdown of miR-145-3p decreased the expression of some myogenic differentiation markers (MyoD, MyoG, MYHC, Myomaker, and Myomerger) during MuSC differentiation. Statistical significance differences (*p* < 0.05) were represented with *, *p* < 0.01 by **, and *p* < 0.001 by ***. (**D**) Knockdown of miR-145-3p inhibited myotube formation. MuSCs were differentiated for 5 days, transfected with LNAantimiR-145-3p, and stained with anti-DAPI and anti-MyHC (green). Immunofluorescence images were captured using a fluorescence microscope (Olympus, Tokyo, Japan) (scale bar = 100 µm), and the results were determined using the software Image J. (**E**,**F**) Image J software was employed to count cells, and the % MyHC+ cell was calculated as the ratio of the number of nuclei surrounded by MyHC signal to the total nuclei. (**G**–**K**) Overexpressing miR-145-3p using AgomiR-145-3p or miR-145-3p mimics increased the mitochondrial mass of active MuSCs, while inhibiting it with LNAantimiR-145-3p or miR-145-3p inhibitor reversed this effect. All experiments had six biological repeats, and statistical significance differences (*p* < 0.05) were represented with *, *p* < 0.01 by **, *p* < 0.001 by *** and *p* < 0.0001 by ****.

Moreover, the knockdown of miR-145-3p inhibited MuSC differentiation (Figure 4). miR-145-3p knockdown reduced the expression of some myogenic differentiation markers (MyHC, MyoG, MyoD, Myomerger, and Myomaker) (Figure 4A–C), inhibited MyHC immunofluorescence (Figure 4D–F), and myotube formation, indicated as the percentage of MyHC-positive cells and the number of 3–6 nuclei myotubes (*p* < 0.05), as shown in (Figure 4E,F). Additionally, ectopic miR-145-3p increased while deficiency of miR-145-3p decreased mitochondrial mass in differentiating MuSCs (Figure 4G–K).

### 2.4. The Knockdown of MYBL1 Simulates the miR-145-3p’s Inhibition on MuSCs Proliferation

**Figure 5:**
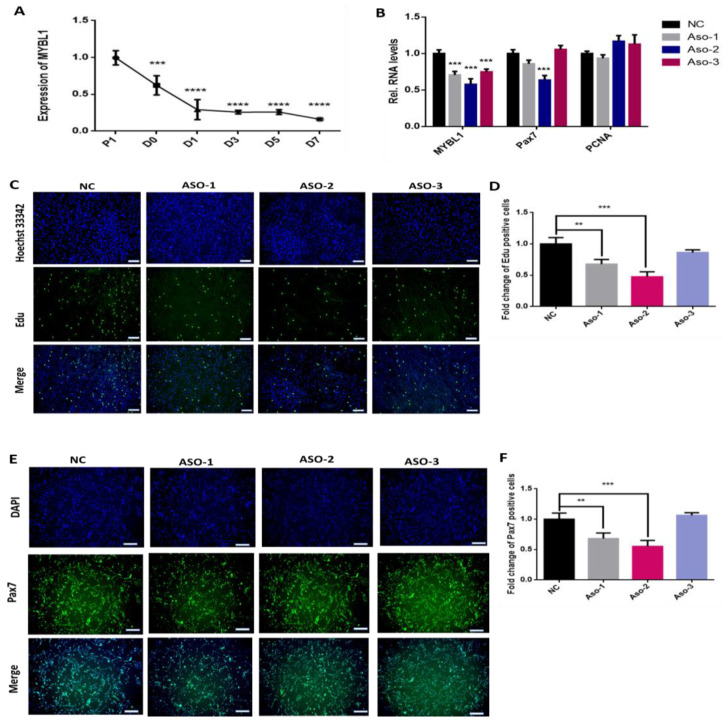
Knockdown of MYBL1 inhibited MuSC proliferation. (**A**) The expression profile of MYBL1 during MuSC myogenesis. (**B**) Knockdown of MYBL1 inhibited the expression of the proliferation markers Pax7 and PCNA. (**C**,**D**) The number of EdU-positive cells and (**E**,**F**) Pax7 immunofluorescence was decreased when MuSCs were transfected with MYBL1-ASo-2′MOE siRNAs. Cell nucleus was stained with Hoechst 33342 and DAPI (blue), proliferating cells with EdU (green), and Pax7 protein with green. The images were captured using a fluorescence microscope (Olympus, Japan) (scale bar = 200 µm), and the results were determined using the software Image J. Graphs were drawn per the fold change of Pax7 and EdU-positive cells. All experiments had six biological repeats and statistical significance differences (*p* < 0.05) were represented with *p* < 0.01 by **, *p* < 0.001 by *** and *p* < 0.0001 by ****.

We subsequently explored the function of MYBL1 during myogenesis. MYBL1 was highly expressed during MuSCs proliferation as compared to differentiation (Figure 5A). The knockdown of MYBL1 decreased the expression of the proliferation marker Pax7, but that of PCNA was not evidenced by the result (Figure 5B). MYBL1 knockdown reduced the number of EdU-positive cells in proliferating MuSCs (Figure 5C,D). There was a significant difference (*p* < 0.05) between MuSCs transfected with MYBL1-ASO-2′MOE siRNA 1 and 2 compared to ASO-3 (Figure 5D). Consistently, MYBL1 knockdown with MYBL1-ASO-2′MOE siRNA 1 and 2 decreased the number of Pax7-positive cells in proliferating MuSCs (*p* < 0.05) (Figure 5E,F). These suggest that the knockdown of MYBL1 decreases proliferation, which mimics that of overexpressed miR-145-3p in proliferating MuSCs.

### 2.5. MYBL1 Mediates the Regulation of miR-145-3p on Expression of Vexin, VCPIP1, COX1, and COX2

**Figure 6:**
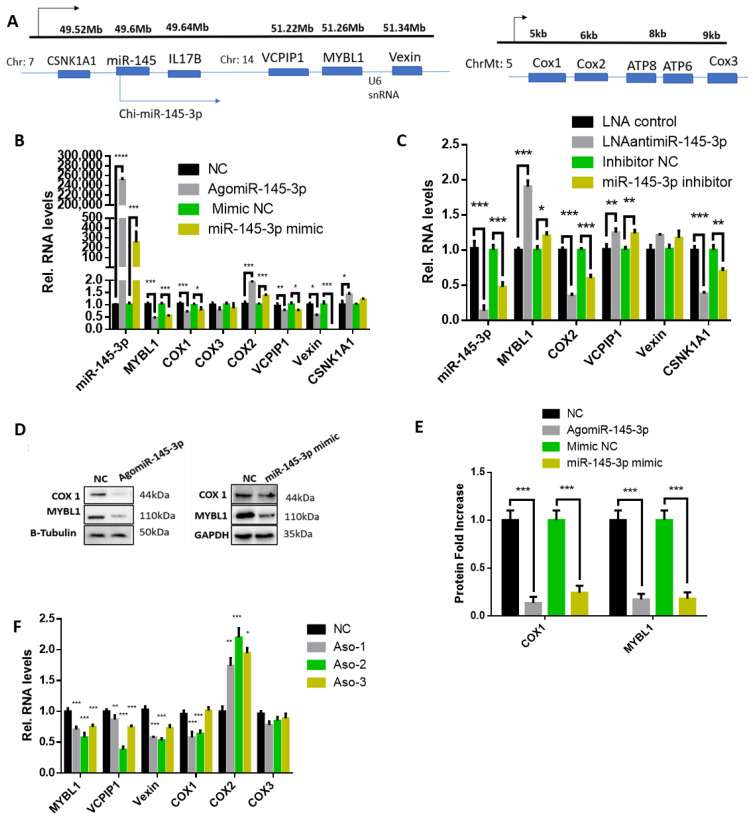
MYBL1 mediated the regulation of miR-145-3p on expression of Vexin, VCPIP1, COX1, and COX2. (**A**) A pictorial representation of miR-145-3p and its neighboring genes (CSNK1A1 and IL17B), MYBL1 and its neighboring genes (VCPIP1 and Vexin), and COX1 and COX3 and their neighboring gene (COX2) on the chromosome [43]. (**B**) Overexpression or (**C**) knockdown of miR-145-3p in proliferating and differentiating MuSCs, respectively, regulates the expression of its neighboring gene (CSNK1A1) and that of MYBL1 (VCPIP1, Vexin) and COX2. (**D**,**E**) Overexpressed miR-145-3p inhibited the protein expression of MYBL1 and COX1. (**F**) Knockdown of MYBL1 reduced the expression of its neighboring genes, VCPIP1 and Vexin, while increasing the expression of COX2 in the mitochondria. All experiments had three biological repeats, and statistical significance differences (*p* < 0.05) were represented with *, *p* < 0.01 by **, *p* < 0.001 by *** and *p* < 0.0001 by ****. Arrows shows the direction of the chromosome and genes from left to right.

Some miRNAs regulate their neighboring genes and those of their target gene [42,43]. Overexpressing miR-145-3p elevated the expression of CSNKA1A, a gene 0.08 Mb downstream of miR-145 in goat chromosome 7, but decreased MYBL1 and its neighboring genes, including VCPIP1 and Vexin (Figure 6A,B). On the contrary, deficiency of miR-145-3p downregulated levels of CSNKA1A, but elevated MYBL1, VCPIP1, and Vexin transcripts (Figure 6C). Meanwhile, the proteins of MYBL1 and COX1, valuated by WB, confirmed that the negative effect of ectopic miR-145-3p using AgomiR-145-3p or miR-145-3p mimic on them (Figure 6D,E). These results confirm that miR-145-3p regulate the gene adjacent to itself and that of target genes during myogenesis.

We explored the regulatory effect of miR-145-3p on some mitochondria genes (COX1, COX2, and COX3) in MuSCs. Overexpressed miR-145-3p decreased the levels of COX1 but increased COX2 (a neighboring gene 1 kb downstream of COX1) in proliferating MuSCs, with little disturb on COX3 (Figure 6A,C). The knockdown of miR-145-3p decreased the expression of COX2 (Figure 6C). These results imply that miR-145-3p regulates COX1 and COX2 genes, which possibly associates with mitochondrial activity.

We subsequently knocked down MYBL1 and found that deficiency of MYBL1 in proliferating MuSCs decreased the expression of VCPIP1, Vexin, and COX1, while that of COX2 was increased (Figure 6F), which exactly mimics the inhibitory effect of ectopic miR-145-3p on these genes. Since miR-145-3p inhibits the expression of MYBL1, the overexpression of miR-145-3p and knockdown of MYBL1 negatively regulated the same set of genes, we speculate that miR-145-3p may regulate these targets via downregulating MYBL1.

## 3. Discussion

MuSCs are located between the plasma membranes of myofibers [2]. After muscle damage, they enter the cell cycle to develop (proliferate and differentiate) multi-nucleated myotubes [3,4]. Myogenesis is regulated by factors, including noncoding RNAs (including circRNAs and miRNAs) and cellular pathways (including PI3K/AKT signaling). miRNAs play crucial regulatory roles during myogenesis [10,11]. miRNAs, including miR-1, miR-133, and miR-206, are expressed in muscle tissues and play roles in muscle development [44].

circRNAs, including CDR1as, regulate the expression of several myomiRs and myogenic mRNAs, hence the knockout or knockdown of these circRNAs distorts this balance [14]. The knockdown of CDR1as in MuSCs resulted in the upregulation of miR-7-5p and miR-145-3p while decreasing the expression of mRNAs, including MYBL1, COX1, and COX3. CDR1as is a competing circRNA that acts as a shield against the negative effects of certain types of miRNAs on the development and expression of mRNAs [45]. Hence, the knockdown of CDR1as in MuSCs could cause the upregulation of certain miRNAs to subsequently decrease the expression of these mRNAs [35].

miR-145-3p is inversely associated with MYBL1 in CDR1as perturbed MuSCs. miR-145-3p and MYBL1 were highly expressed in The cytoplasm, coinciding that miRNAs are located in both the cytoplasm and nucleus [44,46]. Moreover, the regulations in the ratio of miR-145-3p and MYBL1 in both cellular fractions and in CDR1as perturbed MuSCs may imply a possible interaction between these two RNAs [46]. We quantified the luciferase activity in cells transfected with CDR1as and MYBL1 constructs with miR-145-3p, which confirms the sequence-dependent binding of miR-145-3p to MYBL1 and CDR1as transcripts, coinciding with the negative expression correlation between miR-145-3p and MYBL1/CDR1as in proliferating MuSCs. These results establish the inverse relationship between miR-145-3p and MYBL1/CDR1as; the positive correlation between the expression of CDR1as and MYBL1/COX1 imply that CDR1as may sponge miR-145-3p.

The ectopic expression of miR-145-3p decreased myogenic proliferation and the mitochondrial mass of goat MuSCs. miRNAs, including miR-27a, promote myoblast proliferation by targeting myostatin [47]. miR-145-3p reduces proliferation and induces apoptosis and autophagy [48]. In the current study, miR-145-3p inhibited proliferation by inhibiting the expression of myogenic proliferation markers (PCNA and Pax7), reduced Pax7 immunofluorescence, EdU-positive cells, viable MuSCs, and mitochondrial mass. The mass and activity of cellular mitochondria are positively correlated with cellular growth [18,22]. Reduced mitochondrial mass and activity as well as COX expression decreases proliferation and induces apoptosis in myoblast [27,49,50]. Hence, we speculate that decreased MuSC proliferation caused by miR-145-3p may have been achieved via the downregulation of MYBL1 (a proliferation promoter), dysregulated COX expression, and the lowering of MuSC mitochondrial mass during the overexpression of miR-145-3p [49,50,51].

miRNAs, including miR-145-3p, regulate MuSC differentiation. miR-206 promotes myoblasts’ differentiation by targeting Hdac4 and suppressing the expression of DNA polymerase α subunits [11]. Additionally, the inhibition of miR-181 decreased myoblasts’ differentiation [52]. In the current study, the knockdown of miR-145-3p inhibited MuSC differentiation by decreasing the expression of myogenic differentiation markers (MyHC, MyoD, MyoG, and Myomaker), myotube formation, MyHC-positive cells, and mitochondrial mass.

MYBL1 likely mediates the inhibitory effect of miR-145-3p on neighboring genes. miRNAs are known to regulate their neighboring genes and those of their target genes [26,42]. miR-24-1 upregulated the expression of its adjacent genes fructose-1,6-bisphosphatase 1 (FBP1) and Fanconi anemia, complementation group C (FANCC) in HEK293T cells [42]. In this study, miR-145-3p inhibited the expression of VCPIP1 and Vexin (adjacent genes of MYBL1) while increasing the expression of CSNK1A1 (an adjacent gene of miR-145); however, the knockdown of miR-145-3p reversed the situation. Recent studies have shown that the functionality of miRNAs is more complex than previously believed. Multiple novel actions can be performed by small RNAs, including small RNA-based gene activation (TGA) [53]. Hence, an miRNA that inhibits a gene would simultaneously act as an activating miRNA for another, confirming the observations seen in this study with miR-145-3p [46]. MYBL1 promotes proliferation, arrests cell growth, and terminates the differentiation of cells [51]. Intriguingly, the knockdown of MYBL1 mimicked the inhibitory effect of miR-145-3p on VCPIP1 and Vexinand COX1/COX2 in MuSCs. Hence, we speculate that miR-145-3p may regulate these targets via downregulating MYBL1.

Mitochondrial respiration, mass, volume, and copy number increase during myogenic differentiation [54,55]. This causes an increase in mitochondrial enzyme activity, hence drawing the majority of its energy from oxidative phosphorylation. COXs are the core complexes with which nuclear-encoded proteins associate [24]. Therefore, an alteration in the expression of these genes leads to impaired mitochondrial mass and activity [56]. Overexpressed miR-145-3p and/or MYBL1 knockdown inhibited the expression of COX1 but increased that of COX2 in proliferating MuSCs. The alteration in the expression of COX genes could result in changes in mitochondrial mass, activity, respiration, and energy production [57]. This decreases or increases the mitochondria’s energy production and integrity, which might trigger cell death or enhance cell growth and differentiation, respectively [18,27]. This was seen in MuSCs when miR-145-3p was overexpressed or knocked down, resulting in dysregulated COXs expression and mitochondria mass, inhibited proliferation and differentiation, respectively.

## 4. Materials and Methods

### 4.1. Ethics Statement

In this study, all the experimental schemes were approved by the Institutional Animal Care and Utilization Committee of Sichuan Agricultural University, under permit no. DKY-2020202011. As well, they were conducted according to the Regulations for the Administration of Affairs Concerning Experimental Animals (Ministry of Science and Technology, Chengdu, China).

### 4.2. Goat MuSC Isolation and Culture

All cell lines were tested and authenticated prior to the research. MuSCs were isolated from Longissimus dorsi (LD) muscles of Jianzhou big-eared newborn goats [14]. The goats were anesthetized before execution. The excised LD was washed 3 times with 4% phosphate buffer saline (PBS; Merck Millipore, Kankakee, IL, USA) to remove blood from the tissue. It was then disintegrated to about 1 mm^3^ in 500 µL Dulbecco’s Modified Eagle Medium (DMEM) with 4% penicillin, 0.25% trypsin (Thermo Fisher Scientific, Minneapolis, MN, USA) and 50 µL of 0.1% collagenase I (Merck Millipore, Burlington, MA, USA). It was then digested for 1 h. The digest was then sieved using a 100 µM filter and centrifuged at 1000 r/min centrifugation for 5 min. The supernatant was then cultured in growth medium (GM) containing high-glucose DMEM (HyClone, Logan, UT, USA) and supplanted with 10% fetal bovine serum (Gibco, Carlsbad, CA, USA) and 1% antibiotics (Penicillin & Streptomycin (Invitrogen, Carlsbad, CA, USA)). The cells were subsequently digested with trypsin and procured out of the trypsin with the digested fibroblasts to produce a pure culture of skeletal muscle satellite cells while changing the medium and repeating this after 48 h. Finally, MuSCs, after reaching 70–80% confluence, were digested with trypsin and passed into a T75 cell culture tube and later stored in liquid nitrogen for subsequent experiments.

All experiments were performed with mycoplasma-free cells. MuSCs were cultured in GM to induce proliferation. To induce myogenic differentiation, GM was replaced with differentiation medium (DM) containing high-glucose DMEM supplemented with 2% horse serum (HyClone, Logan, UT, USA) and 1% antibiotics after MuSC cells reached 80–90% confluence. The medium was changed every 2 days. MuSCs were cultured at 37 °C and 5% CO_2_.

To verify the authenticity of the isolated MuSCs, cells were morphologically confirmed via microscopic observation and Pax7 immunofluorescence staining. We observed the myogenic proliferation marker Pax7 being positively expressed in the isolated MuSCs. Moreover, myotube formation was evident in the cells after induction of differentiation at day 3 (Supplementary Figure S1). The cells were tested in January 2020 at the Farm Animal Genetic Resources Exploration and Innovation Key Laboratory of Sichuan Province, College of Animal Science and Technology, Sichuan Agricultural University, Chengdu, 611130 China.

### 4.3. RNA Extraction and qRT-PCR

The nuclear and cytoplasmic fractions of cultured MuSCs were extracted using Nuclear Extraction Kit (ab113474) (Abcam, Cambridge, MA, USA). Total RNAs were extracted by TRIzol reagent according to the manufacturer’s protocol (Thermo Fisher Scientific, Waltham, MA, USA). For RNase R treatment, 1 µg of total RNA was incubated for 20 min at 37 °C with and without RNase R (2 µg) and purified using the RNeasy MinElute cleaning Kit (QIAGEN, Hilden, Germany). mRNA reverse transcription was done using the PrimeScriptRT™reagent Kit with gDNA Eraser (Perfect Real Time) (Takara Biomedical Technology Co., Ltd., Beijing, China) following the manufacturer’s protocol. miRNA-specific stem-loop primers were used to reverse-transcribe cDNA from the microRNAs. miRNA reverse transcription was done using the Mir-X™ miRNA First-Strand Synthesis Kit (Takara Biomedical Technology Co., Ltd., Beijing, China) following the manufacturer’s protocol. Subsequently, primers were used for the subsequent qRT-PCR. The 2^−∆∆Ct^ method was used to calculate the expression levels from the qRT-PCR data. Primers used for qRT-PCR can be seen in Supplementary Table S3. 

### 4.4. Validation of RNA-Seq Data

The RNA-seq data from our previous research [35] was validated by quantifying the expression levels of some differentially expressed (DE) upregulated and downregulated miRNAs and mRNAs during CDR1as knockdown, and we compared it to that of the RNA-seq data. The same constructed cDNA used for the RNA-seq [35] was used for the validation via qRT-PCR. The obtained Ct values were normalized to the internal control (GAPDH) and the fold change was calculated.

### 4.5. Quantification of the Expression Profiles of Some Myogenic Regulatory Genes in MuSCs

To quantify the expression of CDR1as during myogenesis, MuSCs (~1 × 10^5^ cells per well) were proliferated (2 days) and differentiated for 7 days with 3 biological replicates. Cells were harvested at intervals of 24 h during proliferation (P1, P2/DO) and 48 h during differentiation (D1, D3, D5, and D7). Total RNA was extracted and used to construct cDNA for qRT-PCR. The expression profile of some myogenic proliferation (PCNA, proliferating cell nuclear antigen; Pax7, paired box 7) and differentiation (MYHC, myosin heavy chain; MyoG, myogenin) genes were quantified via RT-qPCR using them as a positive control. Additionally, the expression profile of other DE mRNAs (STAT2) and DE miRNAs (miR-206, miR-181c, miR-135-5p, and miR-423-5p) were measured via qRT-PCR.

### 4.6. Plasmid Construction

Fragments of CDR1as, MYBL1, COX1, and COX3 3UTRs containing the binding site of miR-145-3p were amplified and inserted into the psiCHECK-2 vector (Promega, Madison, WI, USA) at the 3′ end of the Renilla gene using restriction enzymes Xhol 1 (CTC GAG) and Not 1 (GCGGCCGC) (Takara Biomedical Technology Co., Ltd., Beijing, China) and T4 DNA ligase with an empty vector as a control. The mutant sequence was designed by altering some nucleotides at the complementary binding sequence of miR-145-3p on MYBL1 3UTR and CDR1as. Primers with the site-specific mutation were designed using the NEBaseChanger™ (https://nebasechanger.neb.com/)(accessed on 25 September 2020) and used to clone the mutant sequence from the wild-type sequences (CDR1as and MYBL1 3UTR) following the protocol from the Q5^®^ Site-Directed Mutagenesis Kit Quick (E0554). Trelief ™ 5 alpha Chemical Competently Cell (Tsingke Biotechnology Co., Ltd., Beijing, China) was used for the conversion of recombinant plasmids.

### 4.7. Cell Culture and Treatments

Cultured myoblasts (~1 × 10^5^ cells per well) were transfected with siCDR1as siRNAs, AgomiR-145-3p, miR-145-3p mimics, LNAantimiR-145-3p, miR-145-3p inhibitor, MYBL1-ASO 2′MOE siRNAs (Aso-1, Aso-2 and Aso-3), and their respective controls when MuSCs reached ~80% confluence. MuSCs at 80% to 90% confluence in growth or differentiation medium were replaced with antibiotic-free medium 24 h before transfection. The transfection medium of MuSCs was changed to growth or differentiation medium containing antibiotics 4–6 h post-transfection, and cells were then harvested or prepared for subsequent experiments. The transfection efficiency of AgomiR-145-3p, miR-145-3p mimics, miR-145-3p inhibitor and miR-145-3p LNA in MuSCs can be seen in Supplementary Figure S5.

AgomiRs are modified miRNA agonist which have the same function as miRNA mimics but are highly stable and robust compared to the mimics [58,59]. Locked nucleic acid (LNA) are stable and have modified DNA which enhances their binding affinity to complementary DNA sequences compared to miRNA inhibitors. We used the inhibitory effects of the LNA to design a stable anti-miRNA for this studies [60,61]. In this current study we compared the overexpression effect of miRNA mimics and miRNA AgomiR on miR-145-3p function. We also compared the inhibitory effect on miR-145-3p using miRNA inhibitors and LNA-antimiRNA. This is because studies have confirmed the superiority of AgomiRs and LNA inhibitors over mimics and miRNA inhibitors, hence why we tried to verify these results in the current study [58,59,60,61]. Similar results from previous studies were confirmed in the current study, where the effect of AgomiRs and LNA inhibitors on miR-145-3p was higher compared to mimics and miRNA inhibitors, respectively.

### 4.8. Cell Number Assay

The EdU and CCK-8 assays were performed with different treatment groups (miR-145-3p mimics, AgomiR-145-3p, LNAantimiR-145-3p, miR-145-3p inhibitor with all their respective controls) of cultured and transfected MuSCs. Myoblasts were treated with dimethyl sulfoxide as a control to the medium (Thermo Fisher Scientific, Minneapolis, MN, USA) for the EdU assay. For the EdU assay, MuSCs (1 × 10^5^ cells/well) were seeded in 48-well plates and incubated under standard conditions in triplicates. EdU was performed 24 h post-transfection. After incubation with 50 mM of EdU from the Beyotime Click Edu-488 Assay Kit (Meilunebio, Shanghai, China) for 2 h, cells were fixed and stained for EdU as described in the manufacturer’s protocol. The cell nuclei were counterstained with Hoechst 3342 for 30 min and detected by fluorescence microscopy (Olympus, Tokyo, Japan). For the CCK-8 assay, MuSCs (1 × 10^4^ cells/well) were plated into 96-well culture plates in 100 μL growth medium per well, and each treatment group had 8 independent replicates. The CCK-8 assay was performed at 0 h, 12 h, 24 h, 48 h, and 72 h, respectively, using a CCK-8 kit (Meilunebio, Shanghai, China) following the manufacturer’s protocol.

### 4.9. Luciferase Activity Assay

MuSCs (~1 × 10^5^ cells per well, 37 °C, 5% CO_2_) were seeded into 48-well plates in 6 repeats and were proliferated for 2 days in GM. MuSCs at 80–90% confluence were co-transfected with the constructed vectors (psi-check2-CDR1as-Wild, psi-check2-CDR1as-Mut, psi-check2-MYBL1-Wild, psi-check2-MYBL1-Mut, psi-check2-MYBL1-Mut (pt 616), psi-check2-MYBL1-Mut (pt 675), and psi-check2 empty vector) and miR-145-3p mimics with lip3000 (Thermo Fisher Scientific, Minneapolis, MN, USA). The transfection medium was replaced by GM 5 h post-transfection. The luciferase activity of the transfected cells was measured 48 h post-transfection by lysing and measuring the cellular luciferase activity using the TransDetect Double-Luciferase Reporter Assay Kit (Transgene Biotech, Beijing, China) following the manufacturer’s protocol. The psi-check2 empty vector was used as a negative control. The significance of means differences was analyzed by an unpaired two-tailed *t*-test and presented as mean ± SD with asterisks indicating the significance. Primers and the amplified inserts used for this experiment can be seen in Supplementary Tables S1 and S2.

### 4.10. Immunofluorescence Staining

Proliferating and differentiating (day 4) MuSCs (1 × 10^6^ cells per well) were transfected with either an miR-145-3p mimic, AgomiR-145-3p, LNAantimiR-145-3p, miR-145-3p inhibitor, or their respective controls in 4 independent replications. MuSC were fixed with 4% paraformaldehyde in PBS for 20 min, washed with PBS 3 times, permeabilized with 0.5% Triton X-100 for 10 min, blocked with 5% BSA at 4 °C for 30 min, and then incubated with primary antibody (anti-Pax7 or anti-MyHC diluted 1:200; Abcam, Cambridge, MA, U.S.A) at 40 °C overnight 48 h post-transfection. The cells were subsequently washed three times with PBS and incubated with the corresponding fluorescent secondary antibody (rabbit anti-goat immunoglobulin G [IgG] H & L diluted 1:200 with 1% BSA in PBS; Abcam, Cambridge, MA, USA) at 40 °C for 2 h. Then cells were stained with 5 mg/mL DAPI (4′, 6′-diamidino-2-phenylindole (blue)). Images from at least three or more regions in each well were captured using a fluorescence microscope (Olympus, Tokyo, Japan) and analyzed using Image J software (vision 2.0).

### 4.11. Western Blotting Analysis

The total proteins were extracted from proliferating or differentiating MuSCs (1 × 10^6^ cells per well) transfected with an AgomiR-145-3p, miR-145-3p mimics, LNAantimiR-145-3p, or miR-145-3p inhibitor with their respective controls, using protein lysis radioimmunoprecipitation assay (RIPA) buffer containing 1 mM PMSF (Solarbio, Beijing, China) 72 h post-transfection. Subsequently, proteins in the supernatant were separated by SDS-PAGE, and then transferred to 0.2 mm polyvinylidene fluoride (PDVF) membranes and sealed with 5% skim milk for 2 h at room temperature. After, the membranes were incubated with primary antibodies specific for anti-COX1, anti-MYBL1, anti-PCNA, anti-Pax7, anti-beta-tubulin, anti-Myomaker, anti-MyoD, anti-MyHC, and anti-MyoG (ABclonal Biotechnology Co., Ltd., Hubei, China) at 4 °C overnight. The PVDF membranes were washed with Tris saline with Tween (TBST) buffer 3 times and then incubated with horseradish peroxidase (HRP)-conjugated secondary antibodies for 2.5 h at room temperature. The enhanced chemiluminescence luminous fluid (ECL) was applied for color development.

### 4.12. Mitochondria Mass Assay

Mitochondrial staining was done using the MitoTracker™ Green FM—Special Packaging (Thermo Fisher Scientific, Minneapolis, MN, USA) following the manufacturer’s protocol. Proliferating and differentiating MuSCs (1 × 10^6^ cells per well) were transfected with an AgomiR-145-3p, miR-145-3p mimics, LNAantimiR-145-3p, or miR-145-3p inhibitor with their respective controls in 3 replications each. The medium covering the cells was removed and replaced with freshly prepared, pre-warmed growth or differentiating medium containing prewarmed (37 °C) staining solution containing a MitoTracker^®^ probe 24 h post-transfection. MuSCs were incubated for 15–45 min under growth conditions. Following that, the nucleus was stained with Hoechst 33342 and incubated for 10 min under growth conditions. The staining solution was replaced with fresh prewarmed PBS buffer and then images were captured using a fluorescence microscope (Olympus, Tokyo, Japan) and analyzed using Image J.

### 4.13. Nuclear and Cytoplasmic Extraction

The nuclear and cytoplasmic constituent of proliferating (2 days) and differentiating (5 days) MuSCs (1 × 10^6^ cells per well) were extracted using the NE-PER™ Nuclear and Cytoplasmic Extraction Reagents Kit (Thermo Fisher Scientific, Minneapolis, MN, USA) following the manufacturer’s protocol. Total RNAs extracted from these MuSCs were converted to cDNA (Mir-X™ miRNA First-Strand Synthesis Kit (Takara Biomedical Technology Co., Ltd, Beijing, China.) and PrimeScriptRT™reagent Kit with gDNA Eraser (Perfect Real Time) (Takara Biomedical Technology Co., Ltd., Beijing, China)). The relative RNA expressions of miR-145-3p and MYBL1 were quantified via qRT-PCR, respectively, using the cDNA.

### 4.14. LNAantimR-145-3p and MYBL1-ASO-2′MOE siRNAs Design

LNAanitmiR-145-3p was designed as previously described by [62] and synthesized by (Sangong Bioengineering Co., Ltd., Shanghai, China). The LNA-antimiR molecules were synthesized as unconjugated oligonucleotides with a phosphodiester backbone. The perfectly matching LNA-antimiR oligonucleotide: 5′CamCtaTTtcCaGgAmC3′ [3′attcctggaaatactgttctt5′] (capital letters, LNA; mC, LNA methylcytosine; lowercase letters, DNA) was complementary to nucleotides 2–16 in the mature miR-145-3p sequence. The mismatch LNA control oligonucleotide was synthesized with the following sequence: 5′CamCtaTAtcCaGgTmC3′ [5′aagaacagtatttccaggaat3′].

The MYBL1-ASO-2′MOE siRNA was designed with reference to articles [63,64,65] and synthesized by RiboBio, China. Three ASOs were designed targeting different sites of the MYBL1 gene using the following modification formula:

mN*mN*mN*mN*mN*N*N*N*N*N*N*N*N*N*N*mN*mN*mN*mN*mN*

“*” as phosphorothioate bonds added to the sequence, “m” as 2′-O-Methyl bases, and

“N” as nucleotide bases.

### 4.15. Statistical Analysis

GAPDH (glyceraldehyde-3-phosphate dehydrogenase) was used as an internal control for the RT-qPCR. Data were analyzed by an unpaired two-tailed *t*-test shown as mean ± SD with asterisks indicating the significance. Images were analyzed using image J and graphs were drawn using graph pad Prism.

## 5. Conclusions

**Figure 7:**
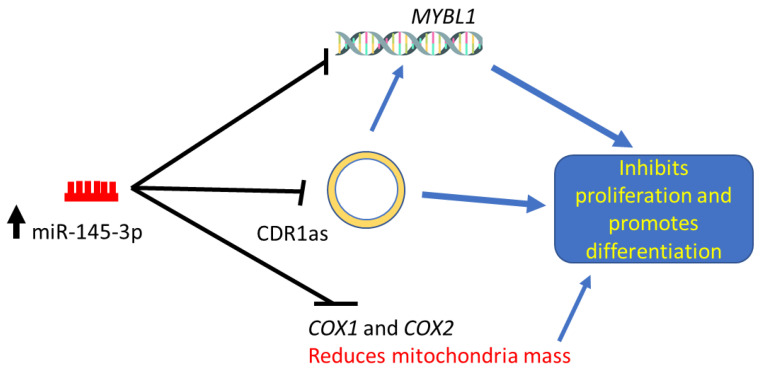
Schematic working diagram of miR-145-3p in MuSCs.

We altered the expression of miR-145-3p, which subsequently regulated MYBL1, CDR1as, COX1 and COX2 expression levels to inhibit MuSC proliferation and promote differentiation. miR-145-3p regulated the expression of COX1 and COX2 directly and/or via downregulating MYBL1 (Figure 7). Further research should explore the role of miR-145-3p and MYBL1 in mitochondrial activities.

## Data Availability

The datasets used and/or analyzed during the current study are available from the corresponding author.

## Supplementary Materials

The following supporting information can be downloaded at: https://www.mdpi.com/article/10.3390/ijms24098341/s1.

## Abbreviations

circRNA	Circular RNA
lncRNA	Long noncoding RNA
MEF2	Myocyte enhancer factor 2
miRNA	Micro RNA
MRFs	Myogenic regulatory factors
Pax:	Paired box
PBS	Phosphate buffer saline
RT-PCR	Reverse Transcription PCR
RT-qPCR	Real-time Quantitative PCR
MuSCs	Skeletal muscle satellite cells
UTR	Untranslated region
LNAantimiR	Lock nucleic Acid anti-miRNA
ASO-2′MOE	Antisense oligonucleotides 2′-O-methoxyethyl
PCNA	Proliferating Cell Nuclear Antigen
MyoD	Myogenic Differentiation 1
MyoG	MyoGenin
MYMK	Myomaker
STAT2	Signal transducer and activator of transcription 2
MYBL1	Myb proto-oncogene like 1
COX	Cytochrome c oxidase subunit
ANOVA	Analysis of Variance
CDR1as	Cerebellar degeneration-related protein 1 antisense RNA
CCK-8	Cell Counting Kit-8
DMSO	Dimethyl Sulfoxide
DAPI	4′, 6′-diamidino-2-phenylindole, blue
MyHC	Myosin heavy chain-embryonic
VCPIP1	Valosin containing protein (p97)/p47 complex interacting protein 1
CSNK1A1	Casein kinase 1 alpha 1
