# Supplementary material for: miR-145-3p Inhibits MuSCs Proliferation and Mitochondria Mass via Targeting MYBL1 in Jianzhou Big-Eared Goats
Source: Int J Mol Sci. 2023 May 6;24(9):8341. doi: 10.3390/ijms24098341 (PMC10179409; doi:10.3390/ijms24098341)
Supplement: Supplementary file 1 [file ijms-24-08341-s001.zip › ijms-2272571-supplementary.pdf]

1.Supplementary data

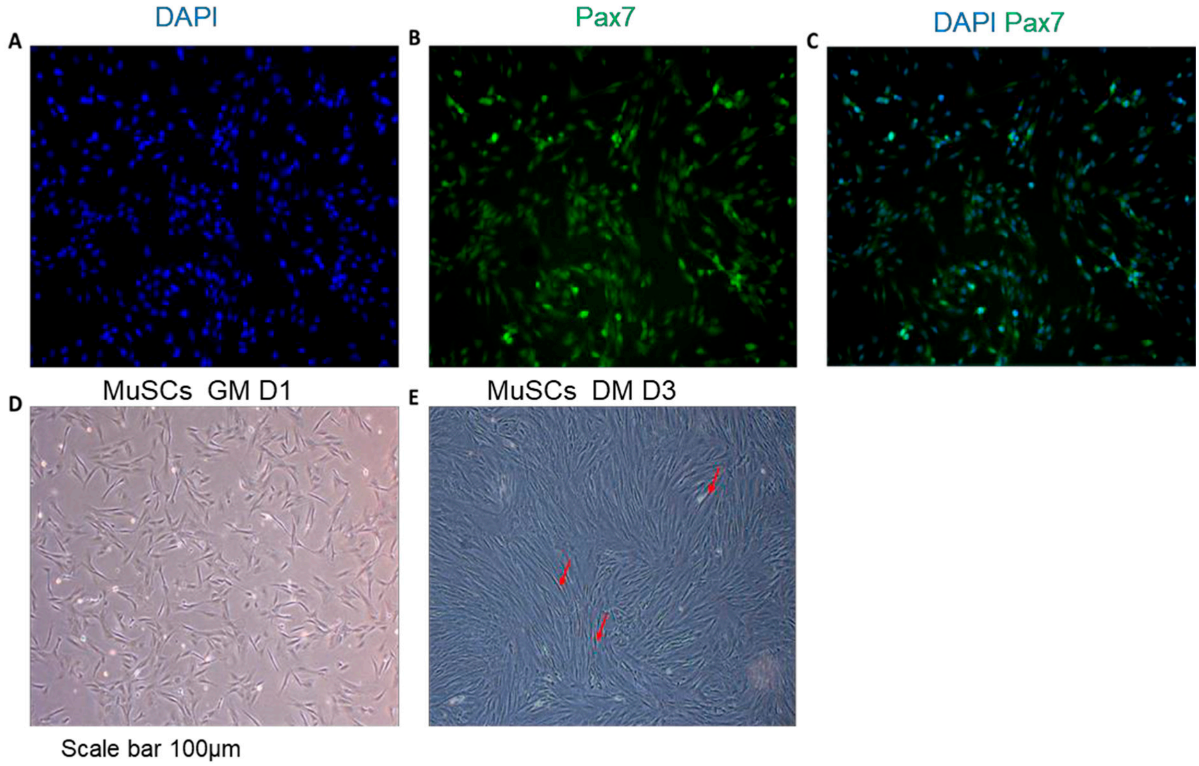

Supplementary Figure S1. Identification of goat SMSCs. (**A- C**) Isolated SMSCs were stained for Pax7 immunofluorescence. The cell nucleus was stained with DAPI (blue) while Pax7 protein was stained green. (**D**) An image of proliferating SMSCs at day 1 and (**E**) differentiating SMSCs at day 3 with evidence of myotube formation pointed with red arrows. Scale bar 100μm.

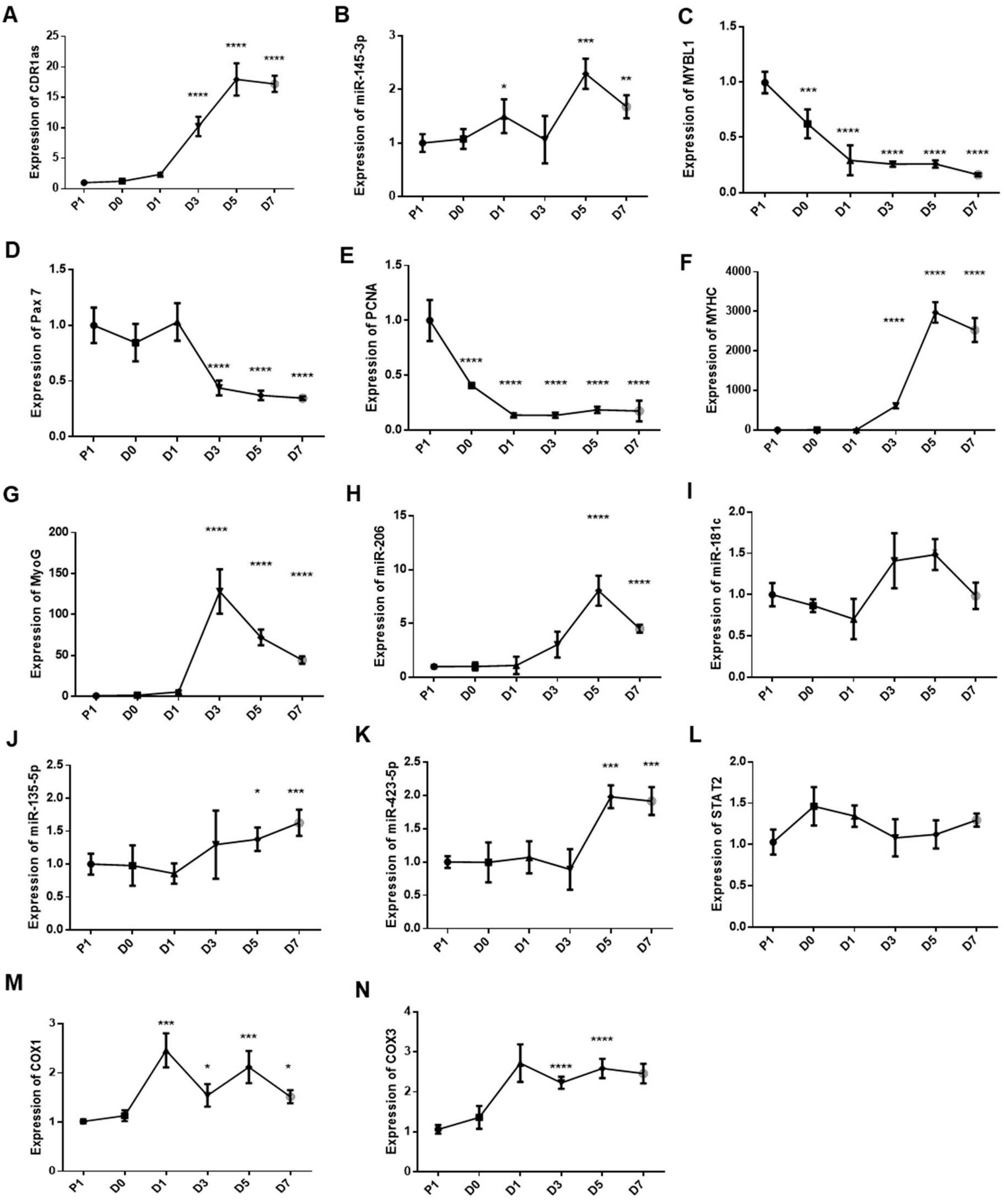

Supplementary Figure S2. The expression profile of some genes and miRNAs during myogenic proliferation and differentiation of goat MUSCs. (**A**) CDR1as, (**B**) miR-145-3p, (**C**) MYBL1, (**D**) Pax7, (**E**) PCNA, (**F**) MyHC, (**G**) MyoG, (**H**) miR-206, (**I**) miR-181c, (**J**) miR-135-5p, (**K**) miR-423-5p, (**L**) STAT2, (**M**) COX1 and (**N**) COX3. All experiments were repeated three times and statistical differences ($P < 0.05$) are differentiated by *, $P < 0.01$ by ** and $P < 0.001$ by ***.

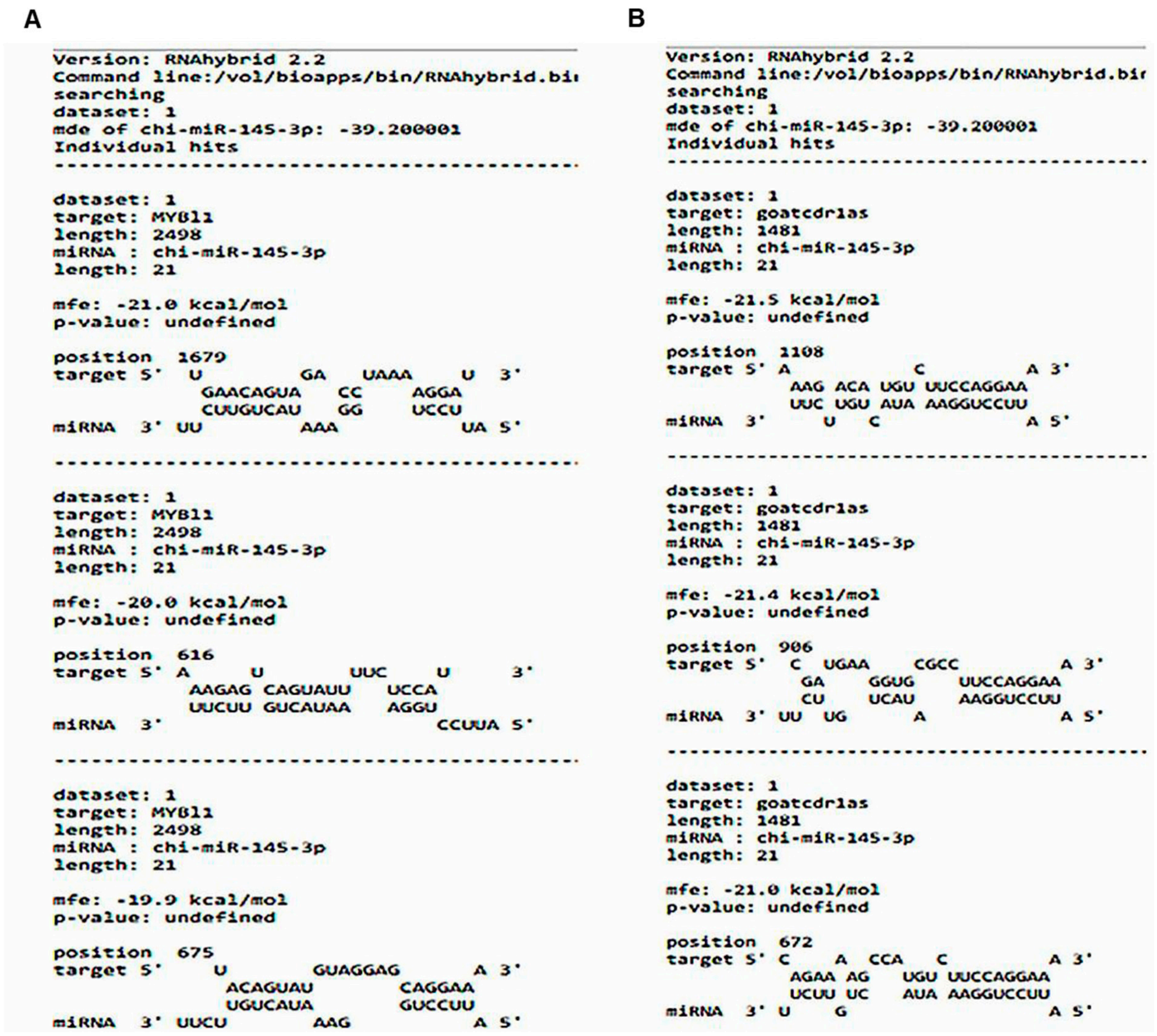

A

```
Version: RNAhybrid 2.2
Command line:/vol/bioapps/bin/RNAhybrid.bi
searching
dataset: 1
mde of chi-miR-145-3p: -39.200001
Individual hits
------------------------------------------

dataset: 1
target: MYBl1
length: 2498
miRNA : chi-miR-145-3p
length: 21

mfe: -21.0 kcal/mol
p-value: undefined

position  1679
target 5'  U        GA   UAAA    U  3'
            GAACAGUA   CC     AGGA
            CUUGUCAU   GG     UCCU
miRNA  3' UU        AAA          UA 5'

------------------------------------------

dataset: 1
target: MYBl1
length: 2498
miRNA : chi-miR-145-3p
length: 21

mfe: -20.0 kcal/mol
p-value: undefined

position  616
target 5' A      U       UUC    U     3'
           AAGAG CAGUAUU    UCCA
           UUCUU GUCAUAA    AGGU
miRNA  3'                       CCUUA 5'

------------------------------------------

dataset: 1
target: MYBl1
length: 2498
miRNA : chi-miR-145-3p
length: 21

mfe: -19.9 kcal/mol
p-value: undefined

position  675
target 5'     U       GUAGGAG      A 3'
               ACAGUAU       CAGGAA
               UGUCAUA       GUCCUU
miRNA  3' UUCU       AAG           A 5'
```

B

```
Version: RNAhybrid 2.2
Command line:/vol/bioapps/bin/RNAhybrid.bi
searching
dataset: 1
mde of chi-miR-145-3p: -39.200001
Individual hits
------------------------------------------

dataset: 1
target: goatcdr1as
length: 1481
miRNA : chi-miR-145-3p
length: 21

mfe: -21.5 kcal/mol
p-value: undefined

position  1108
target 5' A         C          A 3'
           AAG ACA UGU UUCCAGGAA
           UUC UGU AUA AAGGUCCUU
miRNA  3'    U   C             A 5'

------------------------------------------

dataset: 1
target: goatcdr1as
length: 1481
miRNA : chi-miR-145-3p
length: 21

mfe: -21.4 kcal/mol
p-value: undefined

position  906
target 5'  C  UGAA    CGCC          A 3'
            GA    GGUG    UUCCAGGAA
            CU    UCAU    AAGGUCCUU
miRNA  3' UU  UG      A             A 5'

------------------------------------------

dataset: 1
target: goatcdr1as
length: 1481
miRNA : chi-miR-145-3p
length: 21

mfe: -21.0 kcal/mol
p-value: undefined

position  672
target 5' C    A  CCA  C          A 3'
           AGAA AG   UGU UUCCAGGAA
           UCUU UC   AUA AAGGUCCUU
miRNA  3' U    G                  A 5'
```

Supplementary Figure S3. (**A**,**B**) Binding sites of miR-145-3p to MYBL1 3UTR and CDR1as.

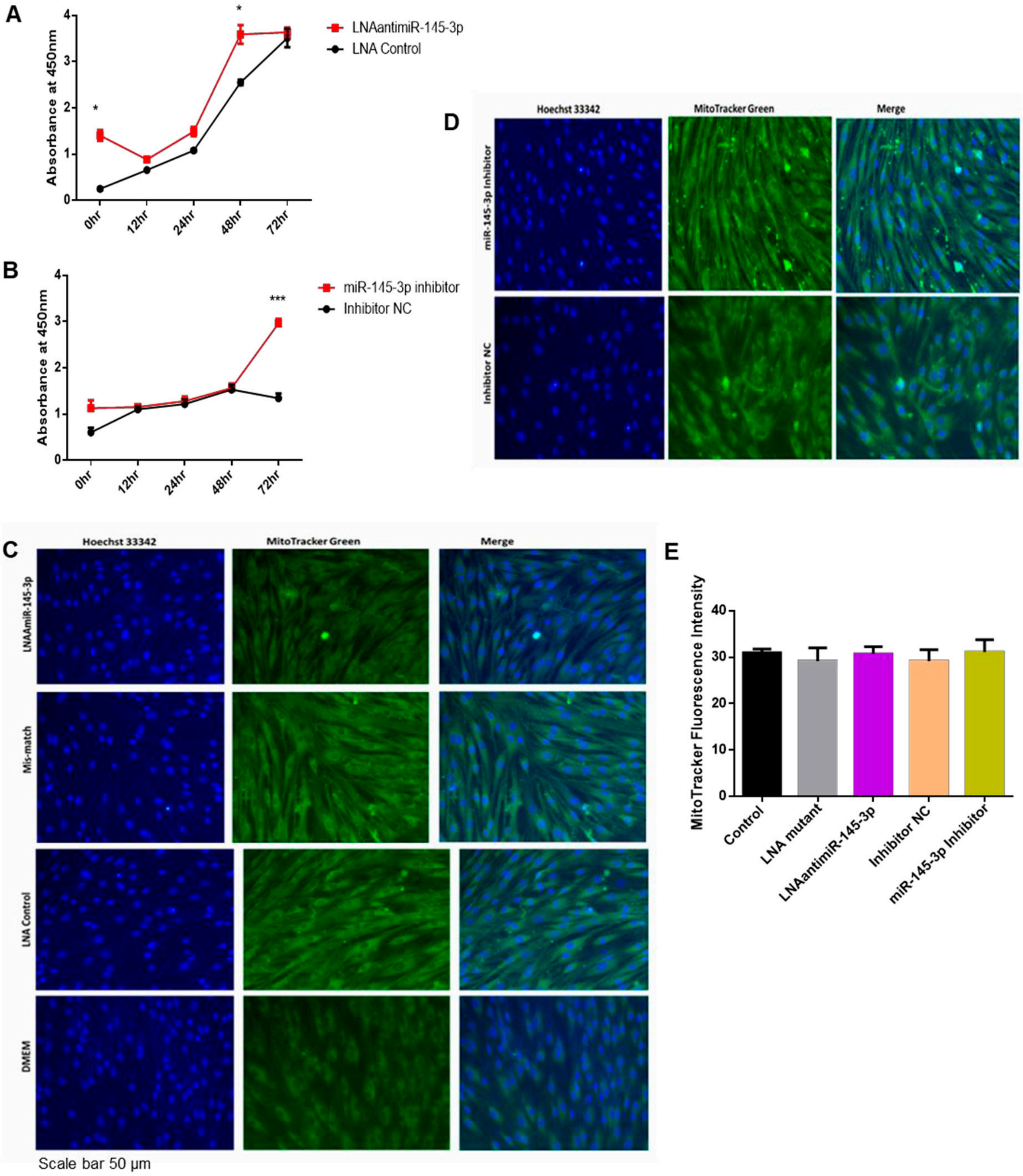

Supplementary Figure S4. Knockdown of miR-145-3p had no significant effect on the number of viable cells and active mitochondria in proliferating MuSCs. (**A-B**) Knockdown of miRNA did not influence cell viability or (**C- D**) active mitochondria numbers. SMSCs were cultured and the nucleus was stained with Hoechst 33342 while the mitochondria were stained with MitoTarcker green (**E**). Scale bar 50 μm. All experiments were repeated three times and statistical differences ($P < 0.05$) are differentiated by *, $P < 0.01$ by ** and $P < 0.001$ by***.

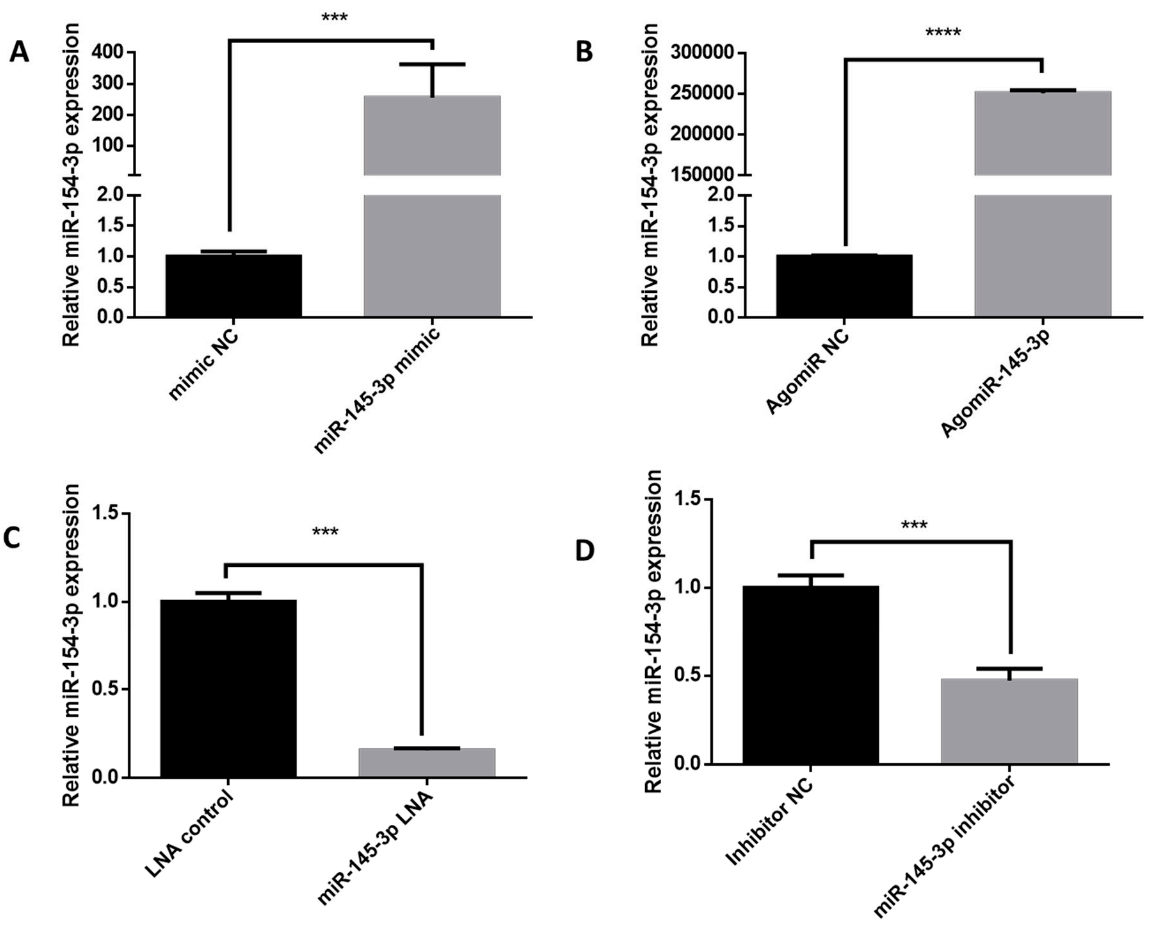

Supplementary Figure S5. The transfection efficiency of (**A**) AgomiR-145-3p, (**B**) miR-145-3p mimics, (**C**) miR-145-3p inhibitor and (**D**) miR-145-3p LNA in MuSCs. All experiments were repeated three times and statistical differences (P < 0.001) are differentiated by ***, P < 0.0001 by ****.

Supplementary Table

Table S1. Primers used for the Dual-luciferase amplification.

Gene	Primer sequence	Product length/bp	Tm (oC)
circ-CDRlas	F CCGCTCGAGCAGAAAAGCCATGTCTTC R AAATATGCGGCCGCATTTCCTGGAAGACATG T	456	63.8
s1-MYBL1	F CCGCTCGAGAAAATGTTTTTTCCTTATT R AAATATGCGGCCGCGATTTCTTTCCAATTTAC A	126	58.8

ck-1-MutMYBL1	F TAACTCGTTGACGCCTTCTTT<br>R TTTCTTTCCAATTTACAAAG	150	49.4
1-2MutMYBL1	F GCCTTCTTTATAAATCTCAGTGAGCTGAG<br>R<br>GTCAACGAGTTACCAATAAGGAAAAAACATT TTC	122	51.5
2-1mutMYBL1	F<br>GCCGCGTTCAAACTTTGTAAATTGGAAAGAA ATC<br>R<br>GTCAACGAGTACAATACATTCAACAAAACTC TC	150	60.3
chk2mutMYBL1	F ATGTTTTTTCCTTATTGGTAA<br>R TTTGAACGCGGCGTCAACGAGTACA	200	53.5
1-1MutMYBL1	F<br>TTTTCCTTATTGGTAACTCGTTGACGCCTTCTT TAT<br>R<br>GCTCACTGAGATTTATAAAGAAGGCGTCAAC GAGTT	170	50.3
1,2chkmutMYBL1	F TTCCTTATTGGTAACTCGTTG<br>R TTTACAAAGTTTGAACGCGG	500	59.0
3utr-MYBL1	F AAAGAGTCAGTATTTTCT<br>R AATCCTTTTAGGTCTACT	1086	44.7

Table S2. Inserted sequence of dual-luciferase reporter vectors.

Name	Sequence
CDR1as wild	ccgctcgagCAGAAAAGCCATGTCTTCCAGGAAATCCATGTCTTCCATCAAATCCAT<br>GGCTTCCAGAAAATCAATGTCTTCCAGGAAATCCATGTCTTCCAGCAAATATTT<br>GTCTTCCAACAAAACTGTGTCTTCCATCAAATTCATGTCTTCCAGCCTATCCATC<br>TCTTCCAGAAAATCCTCGTCTTCCAATGAAGGTGCACCTTCCAGGAAATCCACG<br>TCTTCCAGAAAATCTTCGTCTTCCGATGAAGGTGCGCCTTCCAGGAAATCCACG<br>TCTTCCAGAAAATCCTAGTCTTCCAATGAAGGTGCGCCTTCCAGGAAATCCACG<br>TCTTCCAGAAAATCCTCGTCTTCCAATGAAGGTGCACCTTCCAGGAAATCCATG<br>TCTTCCAGAAAATCCCTGTCTTCCAATGAAGGTGCGCCTTCCAGGAAATCCTTG<br>TCTTCCAACAAAGACATGTCTTCCAGGAAAaaatatgcggccgc
CDR1as mutant	ccgctcgagCAGAAAAGCCATGTCGCATCTAGCATCCATGTCTTCCATCAAATCCAT<br>GGCTTCCAGAAAATCAATGTCGCATCTAGCATCCATGTCTTCCAGCAAATATTT<br>GTCTTCCAACAAAACTGTGTCGCATTCAAATTCATGTCTTCCAGCCTATCCATCT<br>CTTCCAGAAAATCCTCGTCTTCCAATGAAGGTGCACCGCATCTAGCATCCACGT<br>CTTCCAGAAAATCTTCGTCTTCCGATGAAGGTGCGCCGCATCTAGCATCCACGT<br>CTTCCAGAAAATCCTAGTCTTCCAATGAAGGTGCGCCGCATCTAGCATCCACGT<br>CTTCCAGAAAATCCTCGTCTTCCAATGAAGGTGCACCGCATCTAGCATCCATGT<br>CTTCCAGAAAATCCCTGTCTTCCAATGAAGGTGCGCCGCATCTAGCATCCTTGT<br>CTTCCAACAAAGACATGTCGCATCTAGCAaaatatgcggccgc
MYBL1 3UTR wild	ccgctcgagAAAATGTTTTTTCCTTATTGGTAAAGAGTCAGTATTTTCTCCATAAATC<br>TCAGTGAGCTGAGAGTTTTGTTGAATGTATTGTACAGTATGTAGGAGCAGGAA<br>AACTTTGTAAATTGGAAAGAAATCTGTTTTTATAATTTATTTTCATTTTTAAAGC<br>TTAAATGTAGATATTTAAACTTATACAGGGTGTCTAGAAGCCAATGTTGTTTCC

	TGTTATTACAGATAACATAGTAAAGAACAATTTTGACTTTTAAGTATGAAACAG<br>TAGTAAGTTATAGCTGCAAAGAATTCAGTATCTATACTGTATGTCACATCTACC<br>TAAATATTGCACTATGTCCTTTAAATCATGTTGGTTATAAAGTAGTTCTAAAAAT<br>TTACTAAATAATAATTTAATATTTTCTTTTTAAATTATATGGGGGTCATATAAA<br>TTAATCTGGTGATTTGTATATTTGTTTTAAATTTTTGCATTTTGTTTAAAAAAATA<br>ATATGGTACTTTGGTCCCTAAAAACAGTCTGCACTTAGAAGTTTATTATATTTAC<br>TCAGTGTTTAAGAAGTAGAGAACAGTATCTTTTATTTATAAAAATATTTGTCCTT<br>TTATAAATGTTTTGTGTTTCTCTACAGGTTACAACAGTTGCTTCAGTTGCCTGTTT<br>TAGGTGTTTGCACTTACTTTATTTCTTCTTGAAAGAATGTCTTTATTTGCTTTTGT<br>GTAGAGATTTTATGTAATTTTTTGAAACATATAATGGTATGCTGTCAACTTAAA<br>CACTGACAGGTAAATAGAATTGTACACTGTAGTTGTAATTATTTGTAATTGACA<br>CACTCTCTCCCTCTCCACTCCTGAAGTATGCTGCTATAGAAAATAGCAGAATCG<br>GCTTGCTGCTATGAGAGAAGGAAAGAGCGACCACCACTTGCACTGTGTGAAAA<br>CATAAAAAGTATGATGATGGCAAGTTCTCAAGTTAACTTAATGGAATCAACCA<br>TTACCAGGCAAATTCTTGCAAATACCAAAATGCTCTGCCTTAAAACAAAAACA<br>GCTTAAGATTCTCTGTTGATGGAAATGGAAATACGAAGTATTTTAATGCCTAGC<br>TTTTGAACAGTAGACCTAAAAGGATTaaatatgcggccgc
MYBL1 3UTR mutant	ccgctcgagAAAATGTTTTTTCCTTATTGGTAACTCGTTGACGCCTTCTTTATAAATCT<br>CAGTGAGCTGAGAGTTTTGTTGAATGTATTGTACTCGTTGACGCCGCGTTCAAA<br>CTTTGTAAATTGGAAAGAAATCaaatatgcggccgc
MYBL1 3UTR mutant (pt 616)	ccgctcgagAAAATGTTTTTTCCTTATTGGTAACTCGTTGACGCCTTCTTTATAAATCT<br>CAGTGAGCTGAGAGTTTTGTTGAATGTATTGTACTCGTTGACGCCGCGTTCAAA<br>CTTTGTAAATTGGAAAGAAATCaaatatgcggccgc
MYBL1 3UTR mutant (pt 675)	ccgctcgagAAAATGTTTTTTCCTTATTGGTAACTCGTTGACGCCTTCTTTATAAATCT<br>CAGTGAGCTGAGAGTTTTGTTGAATGTATTGTACTCGTTGACGCCGCGTTCAAA<br>CTTTGTAAATTGGAAAGAAATCaaatatgcggccgc

Capital letters represent the actual sequence and the smaller letters represent the recognition sites of the Enzyme Not1 and Xhol1.

Table S3. Primers used for qRT-PCR amplification.

Gene	Primer sequence	Product length/bp	Tm (oC)
CDR1as (Divergent primer) (XM_013976302.1)	F AGCAGGCTCAATATCTACG<br>R CTGGAAGACCTTGACACTG	118	53.9
COX-1	F CCTCCTTTCACTTCCTGTATT<br>R TGTGTTTAGGTTTCGGTCTGT	67	53.7
COX-2	F TAGAGGTAGATAACCGAGT<br>R GAACGATTGGTATGAAAC	225	49.5
COX-3	F GAAGGAGACCGTAACCACATA<br>R GGACAACGCAGAGAATCAAAC	175	55.6
CSNK1A1 (XM_005683156.3)	F TTATTCCTTATTGATTTTGGTCC<br>R TTGCCTTGTCCTGTTGTCT	57	54.4
GAPDH (XM_005680968.3)	F GCAAGTTCCACGGCACAG<br>R GGTTCACGCCCATCACAA	249	59
MEF2C (NM_001314204.1)	F CCCCAGTGGTTTCCGTAGCA<br>R GCTGTTGTTGCCAGCCAGTT	183	58.5

miR-1271-3p	AGTGCCTGCTATGTGCCAGG	20	59.5
miR-129-5p	CTTTTTGCGGTCTGGGCTTGC	21	59.5
miR-135-5p	GGTCCTCAGTGTAGCCCAAG	20	59.5
mir-143-3p	TGAGATGAAGCACTGTAGCTCG	22	50
miR-145-3p	ATTCCTGGAAATACTGTTCTT	21	49.8
miR-16	TAGCAGCACGTAAATATTGGCG	22	50.8
miR-181c	CAAGGGTTTGGGGGAACATTCA	21	51.7
miR-218	TGTTTAATATTTTGCAATAATT	22	57.7
miR-379-5p	TGGTAGACTATGGAACGTAGG	21	55.6
miR-423-5p	TGAGGGGCAGAGAGCGAGACT	21	61.5
MYBL1 (XM_005689032.3)	F CAGCAGAACTCACCGACAAAG<br>R GTAGAGAGGACAACACAGCGT	61	57.8
MyHC (XM_013972193.2)	F CGCCCACCTGGAGCGGATGA<br>R CTTGCGGTCCTCCTCGGTCTGGT	171	61.3
MyoD (XM_018058990.1)	F GTGCAAACGCAAGACGACTA<br>R GCTGGTTTGGGTTGCTAGAC	128	60.7
MyoG (NM_001285733.1)	F GGACCCTACAGATGCCCACAA<br>R TTGGTATGGTTTCATCTGGG	101	60.7
Myomaker (XM_018056156.1)	F CCCTGGCTCTCATGTTGCGCTT<br>R TGCACTTCCGGCCTTCTTGTTG	131	56.9
Myomerger (NC_030823.1)	F GGGCTGTCTGTTGTTCGTCC<br>R AGCATTTCAGGGGGCACAGC	146	58.3
Pax7 (XM_018054746.1)	F AGGACGAAGCGGACAAGAA<br>R TCCAGACGGTTCCCTTTGT	91	50.5
PCNA (XM_005688167.3)	F TGAAGAAAGTGCTGGAGGCG<br>R TTTGGACATGCTGGTGAGG	156	61.3
PDE4B (XM_018045239.1)	F GACTTCATCGTCCATCCGCTG<br>R TGGGAGGGGCTCTGGTGTAT	128	59.5
SRSF11	F TGTCTCGTTCAAAATCCCCT<br>R TTCATCGTAGTCCCGTGTAAG	156	54.5
VCPIP1	F TGACCCTCTGTTTGTACCGC<br>R CGATGAAGCACATTGGCGAG	84	60
Vexin	F AGTCCAGCCAGAAGTAGAGT<br>R CTTGGTGAAGAGATGTTGAGAG	79	55.6
U6	F CTCGCTTCGGCAGCACA<br>R AACGCTTCACGAATTTGCGT	94	55.9
GAPDH (XM_005680968.3)	F GCAAGTTCCACGGCACAG<br>R GGTTCACGCCCATCACAA	249	59.0
